# Troponin predicts mortality in heart failure-related cardiogenic shock

**DOI:** 10.1007/s11739-025-04197-7

**Published:** 2025-11-18

**Authors:** Benedikt N. Beer, Jonas Sundermeyer, Lisa Besch, Angela Dettling, Marvin Kriz, Paulus Kirchhof, Stefan Blankenberg, Letizia Bertoldi, Jeroen Dauw, Ralf Westenfeld, Patrick Horn, Matthew Kelham, Peter Luedike, Enzo Luesebrink, Martin Orban, Clemens Scherer, Norman Mangner, Nuccia Morici, Luca Villanova, Marek Sramko, Michal Pazdernik, Alastair Proudfoot, Robert H. G. Schwinger, Antonia Wechsler, Matthias Pauschinger, Dennis Eckner, Tobias Graf, Octavian Maniuc, Peter Nordbeck, Sven Moebius-Winkler, Carsten Skurk, Holger Thiele, Dirk Westermann, Benedikt Schrage

**Affiliations:** 1 Department of Cardiology, University Medical Center Hamburg-Eppendorf, Martinistr. 52, 20246 Hamburg, Germany; 2 German Centre for Cardiovascular Research (DZHK), Partner Site Hamburg/Luebeck/Kiel, Germany; 3 Department of Clinical Science and Education, Södersjukhuset, Karolinska Institutet, Stockholm, Sweden; 4 Institute of Cardiovascular Sciences, University of Birmingham, Birmingham, UK; 5 Center for Population Health Innovation (POINT), University Medical Center Hamburg-Eppendorf, Hamburg, Germany; 6 Cardio Center, Humanitas Clinical and Research Center - IRCCS, Rozzano, Milan Italy; 7 Cardiovascular Center Aalst, OLV Hospital, Aalst, Belgium; 8 Department of Cardiology, Pneumology and Angiology, Duesseldorf University Hospital, Duesseldorf, Germany; 9 Barts Heart Centre, North Middlesex University Hospital NHS Trust, London, UK; 10 Department of Cardiology and Angiology, University Hospital Essen, Essen, Germany; 11 Department of Medicine I, University Hospital, LMU Munich, Munich, Germany; 12 Department of Internal Medicine and Cardiology, University Hospital Carl Gustav Carus Dresden, Dresden, Germany; 13 IRCCS S. Maria Nascente - Fondazione Don Carlo Gnocchi ONLUS, Milan, Italy; 14 ASST Grande Ospedale Metropolitano Niguarda, Milan, Italy; 15 Department of Cardiology, IKEM, Prague, Czech Republic; 16 Department of Perioperative Medicine, Barts Heart Centre, St. Bartholomew’s Hospital, London, UK; 17 Department of Internal Medicine II, Klinikum Weiden, Weiden, Germany; 18 Department of Internal Medicine 8, Cardiology, Klinikum Nuremberg, Nuremberg, Germany; 19 University Heart Center Luebeck, University Hospital Schleswig-Holstein, Luebeck, Germany; 20 Department of Internal Medicine I, University Hospital Wuerzburg, Würzburg, Germany; 21 Department of Internal Medicine I, University Hospital Jena, Jena, Germany; 22 Department of Cardiology, Angiology and Intensive Care Medicine, Deutsches Herzzentrum Der Charité (DHZC), Campus Benjamin Franklin, Berlin, Germany; 23 German Centre for Cardiovascular Research (DZHK), Partner Site, Berlin, Germany; 24 Department of Internal Medicine and Cardiology, Heart Center Leipzig at University of Leipzig and Leipzig Heart Science, Leipzig, Germany; 25 Department of Cardiology and Angiology I, University Heart Center Freiburg, Bad Krozingen, Germany

**Keywords:** Cardiogenic shock, Troponin, Biomarker, Risk prediction, Mortality, Heart failure

## Abstract

Background: In patients with cardiogenic shock (CS), predicting risk of mortality may improve treatment allocation beyond intensive care admission and thereby outcomes. Troponin appears to be a suitable biomarker but has primarily been evaluated in the setting of infarct-related CS, not in heart failure-related CS (HF-CS), which accounts for almost 50% of cases. *Objectives:* To assess the association of Troponin T with shock severity and mortality in HF-CS patients. *Methods:* Heart failure-related CS patients treated in 15 tertiary care centres (5 European countries, 2016–2021) were retrospectively enrolled (NCT03313687). Association of baseline high-sensitive Troponin T and its 24-h kinetics with shock severity according to the SCAI classification and with in-hospital mortality was assessed by fitting multivariable adjusted regression models. *Results:* *N* = 477 patients (mean age 62 years, 30.2% women). High-sensitive Troponin T at baseline (median 164 ng/l) was significantly associated with in-hospital mortality (HR 1.008, 95%CI 1.002–1.013, *p* < 0.01). Increasing Troponin within 24 h from baseline indicated a 2.4-fold higher risk of death vs. decreasing Troponin levels (HR 2.439, 95% CI 1.070–5.558, *p* = 0.03). In addition, higher Troponin T levels correlate with higher SCAI stages (e.g., baseline Troponin T per 250 ng/l increase: OR 5.268, 95%CI: 1.637, 16.953, *p* < 0.01 for SCAI stage D vs. C). *Conclusions:* Troponin T, a marker of myocardial injury, associates with shock severity in patients with heart failure-related CS. It predicts mortality both with its baseline value as well as with its 24-h kinetics. Thus, Troponin may be a suitable marker to guide therapy or clinical trial enrolment in these patients.

**Graphical abstract:**

Central Illustration. Baseline Troponin and its 24-hour kinetics predict all-cause in-hospital mortality in patients with heart failure-related cardiogenic shock. In addition, Troponin associates with shock severity according to the SCAI classification. SCAI: Society for Cardiovascular Angiography and Interventions.

**Figure:**
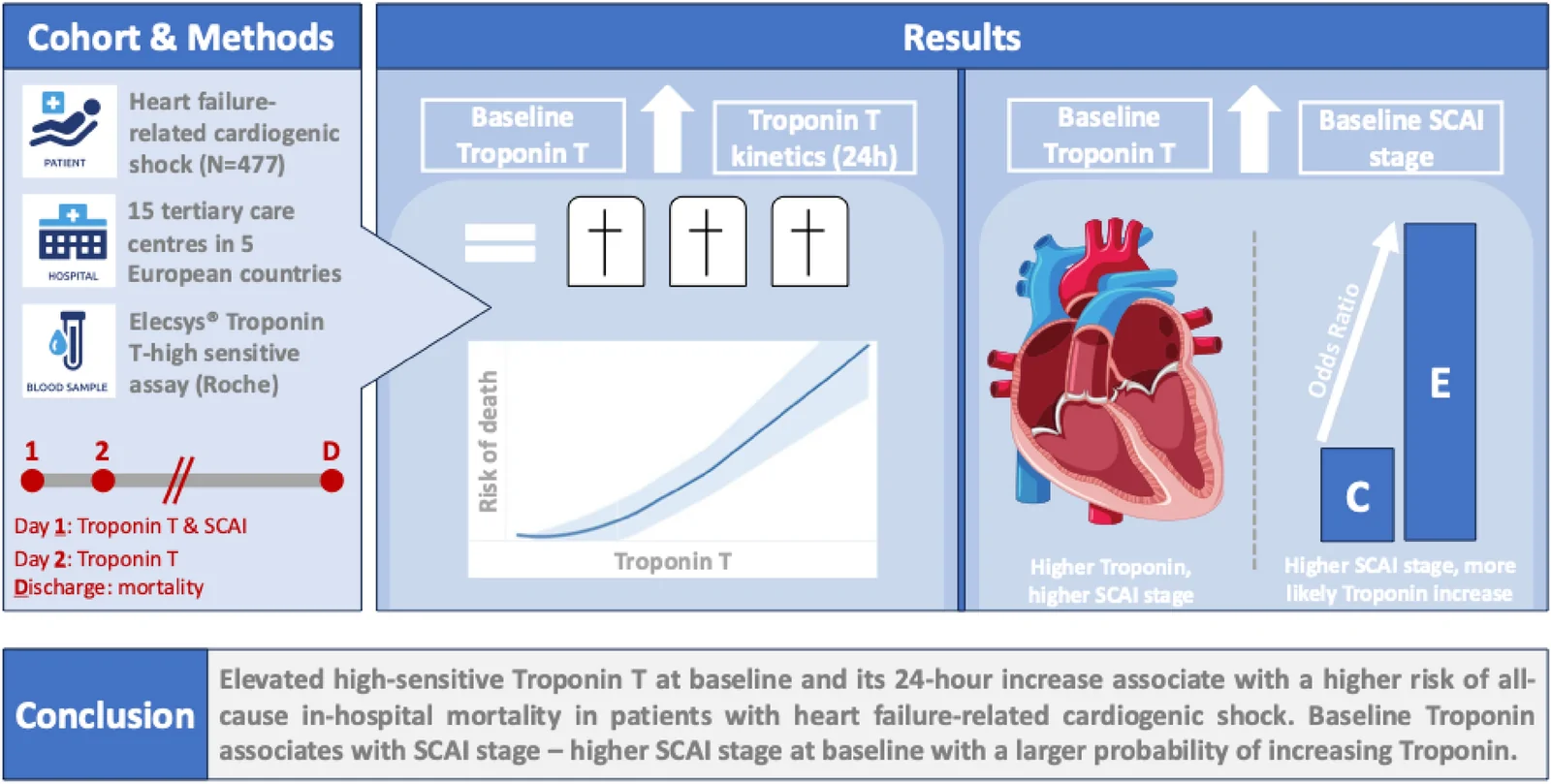

**Supplementary Information:**

The online version contains supplementary material available at https://doi.org/10.1007/s11739-025-04197-7.

## Introduction

Cardiogenic shock (CS) is a syndrome characterised by decreased cardiac output resulting in reduced tissue perfusion and potentially multi-organ failure, with the Society for Cardiovascular Angiography and Interventions (SCAI classification) as a contemporary staging tool [1–3]. CS complicated by acute myocardial infarction (AMI-CS) accounts for approximately half of all CS cases, with decreasing relative incidence, while heart failure-related CS (HF-CS) exhibits an increasing incidence [4]. HF-CS is particularly challenging due to its heterogeneity; differences between relevant strata, like acute on chronic vs. de-novo HF-CS, warrant further investigation [5]. HF-CS only excludes AMI but not prior ischaemic cardiomyopathy, hereby increasing heterogeneity of this condition. Overall in-hospital mortality remains high at approximately 50–60% [4, 6, 7]. Decreasing mortality was shown for AMI-CS but not for HF-CS [4]. This reflects the success of revascularisation of the culprit coronary lesion in AMI-CS patients [1, 7, 8]. There is no such treatment for HF-CS patients, including few and contradicting evidence on the use of mechanical circulatory support devices [1, 7–9]. Randomised controlled trials have mostly been conducted in AMI-CS patients and are, thus, not generalisable to the overall CS population [10]. Positive inotropic and vasoactive drugs may support the failing myocardium, especially in HF-CS patients. However, adverse drug reactions require careful indication by the identification of suitable patients [11]. Moreover, easy-to-use clinical tools for risk prediction were mostly derived from AMI-CS patients, with the exception of the CardShock score and the CSS [7, 12, 13]. This gap of evidence has also been affirmed by the Heart Failure Association of the European Society of Cardiology recently [7]. Troponin reflects myocardial injury in HF-CS in contrast to AMI-CS, in which elevated Troponin predominantly (even though not exclusively) reflects the myocardial infarction. While Troponin is known to be a prognosticator in AMI-CS, it is unknown if it is a suitable risk predictor and therapy guidance marker in HF-CS. Therefore, this study aimed to assess Troponin’s association with mortality in HF-CS patients.

## Methods

### Study design and population

Consecutive patients with CS without AMI treated between 1st January 2016 and 31th December 2021 at 15 tertiary care centres in 5 European countries were retrospectively enrolled (retrospective, observational study STOP-SHOCK, NCT03313687). CS was defined at the discretion of the investigators of each site guided by the SCAI classification [2, 3]. AMI was excluded using clinical symptoms, imaging, the electrocardiogram (ECG) and troponin levels as well as dynamics based on the Fourth Universal Definition of Myocardial Infarction [14]. Other exclusion criteria were: CS primarily caused by right heart failure (e.g., acute pulmonary embolism), cardiopulmonary resuscitation without return of spontaneous circulation and thus implantation of a veno-arterial extracorporeal membrane oxygenation device (eCPR; however, post-cardiac arrest CS with return of spontaneous circulation was included), post-cardiotomy cardiogenic shock and age < 18 years. Baseline was defined according to three different settings: For patients treated with mechanical circulatory support devices, baseline was set as time of the first device’s implantation; for outpatients without any device implantation, baseline was set as the time of admission to the hospital; for inpatients without any device implantation, baseline was set as the time of admission to the intensive care unit. All parameters of disease severity (e.g., lactate, pH, left ventricular ejection fraction [LVEF]) were captured at baseline ± 6 h in order to get the worst value for a given parameter. Information on other, non-time-dependent parameters (e.g., cause of cardiomyopathy) was registered throughout the hospitalisation. However, the exact timing of CS onset prior to the defined baseline is not available in the database.

From this overall study population, only those patients with available baseline high-sensitive Troponin T measurements obtained by Elecsys® Troponin T-high sensitive assay (Roche, Basel/Switzerland) were included in order to exclude bias from variation in Troponin T measurements obtained by different assays as well as to exclude bias from different Troponin protein families (e.g., Troponin T vs. Troponin I).

The primary endpoint of this study was all-cause mortality, censored at discharge or after 30 days (whichever event occurred first), hereafter referred to as “in-hospital mortality”. Also, to assess shock severity as a secondary endpoint, associations with Troponin were assessed.

### Statistics

Continuous variables are shown as medians (interquartile range) or means (standard deviation). Binary variables are shown as counts (frequencies after exclusion of missing values).

Chained equation multiple imputation (using the R-package *mice* with 10 imputed data sets) was used to handle missing data, using the following variables: centre of enrolment, baseline Troponin T (continuous), age (> vs. ≤ 64 years, median), sex, baseline lactate (> vs. ≤ 7.3 mmol/l, median), baseline pH (> vs. ≤ 7.29, median), prior cardiac arrest, acute on chronic vs. de-novo heart failure, baseline LVEF (> vs. ≤ 20%, median), baseline creatinine (continuous) and the primary endpoint of time to in-hospital mortality.

For the regression models, baseline Troponin T as well as 24-h Troponin T kinetics were used as predictors, whereas the latter was dichotomised into increasing/stable vs. decreasing Troponin T by defining decreasing Troponin T as a decrease of at least 20%. All regression models were adjusted for age (> vs. ≤ 64 years, median), sex, baseline lactate (> vs. ≤ 7.3 mmol/l, median), baseline pH (> vs. ≤ 7.29, median), prior cardiac arrest, acute-on-chronic vs. de-novo heart failure, baseline LVEF (> vs. ≤ 20%, median) and baseline creatinine.

The association of Troponin T at baseline and Troponin T kinetics with the SCAI CS stage at baseline was investigated by fitting a logistic regression model; the association of Troponin T at baseline, as well as the association of Troponin T kinetics, with in-hospital mortality was analysed by fitting Cox regression models and illustrated by a Kaplan–Meier curve. Results for the association between baseline Troponin T and in-hospital mortality are also displayed as a polynomial spline. The assumptions underlying the logistic regression models were assessed and met. For all regressions, *p* values < 0.05 were considered as statistically significant and all analyses were computed using R version 4.1.2.

## Results

### Patient characteristics

Overall, 477 patients were included. Mean age was 62 years and 144 patients (30.2%) were women. Approximately half of patients (255, 53.6%) presented with a history of heart failure and the majority had heart failure caused by ischaemic cardiomyopathy (135, 60.3%). The median LVEF was severely reduced by 20%. Of note, 196 patients (41.1%) presented with prior or ongoing cardiopulmonary resuscitation. Median lactate was elevated to 7.3 mmol/l. Concerning shock severity, 238 patients (49.9%) were classified as SCAI stage C, 147 patients (30.8%) as stage D and 92 patients (19.3%) as stage E. During their hospitalisation, 446 patients (93.5%) required treatment with vasopressors and any mechanical circulatory support device was implanted in 238 patients (49.9%). Patient characteristics are displayed in Table 1 and, stratified by Troponin levels at baseline and with missingness, in Supplemental Table 1. Serial Troponin measurements, including missingness, are provided in Supplemental Table 2.

**Table 1:** Patient characteristics. *N* = 477. LVEF: left ventricular ejection fraction. CPR: cardiopulmonary resuscitation. ROSC: return of spontaneous circulation. MCS: mechanical circulatory support devices. hsTroponin T: Troponin T measured by Elecsys® Troponin T-high sensitive assay (Roche). SCAI: cardiogenic shock classification by the Society for Cardiovascular Angiography and Interventions, ranging from A (at risk) to E (in extremis). Vasopressors and MCS: positive if used once during the whole hospitalisation. SD: standard deviation. IQR: interquartile range. Relative frequencies calculated excluding missing values.

Demographical data	
Age (years, mean (SD))	62.44 (15.84)
Sex (female) (%)	144 (30.2)

Heart failure characteristics	
History of heart failure (%)	255 (53.6)
Ischaemic cardiomyopathy (%)	135 (60.3)
LVEF (%, median (IQR))	20 (15, 30)
Resuscitation	
CPR (%)	196 (41.1)
Shockable rhythm (%)	112 (58.0)
ROSC (min, median (IQR))	7 (0, 20)

Haemodynamics	
Systolic blood pressure (mmHg, mean (SD))	82.02 (16.93)
Mean arterial pressure (mmHg, mean (SD))	59.05 (12.90)
Heart rate (bpm, median (IQR))	110 (82, 130)
Vasopressors (%)	446 (93.5)
Use of any MCS (%)	238 (49.9)

Laboratory	
pH (median (IQR))	7.29 (7.16, 7.37)
Lactate (mmol/l, median (IQR))	7.3 (4.2, 10.8)
Creatinine (mg/dl, median (IQR))	1.80 (1.33, 2.53)
hsTroponin T (ng/l, median (IQR))	164 (77, 678)

SCAI at enrolment	
SCAI A (%)	0 (0)
SCAI B (%)	0 (0)
SCAI C (%)	238 (49.9)
SCAI D (%)	147 (30.8)
SCAI E (%)	92 (19.3)

Triggers of shock	
Tachyarrhythmia (%)	137 (31.0)
No trigger identified (%)	130 (29.4)
Infection (%)	90 (20.4)
Stress (%)	32 (7.2)
Non-adherence (%)	16 (3.6)

### Troponin and shock severity

**Fig. 1:**
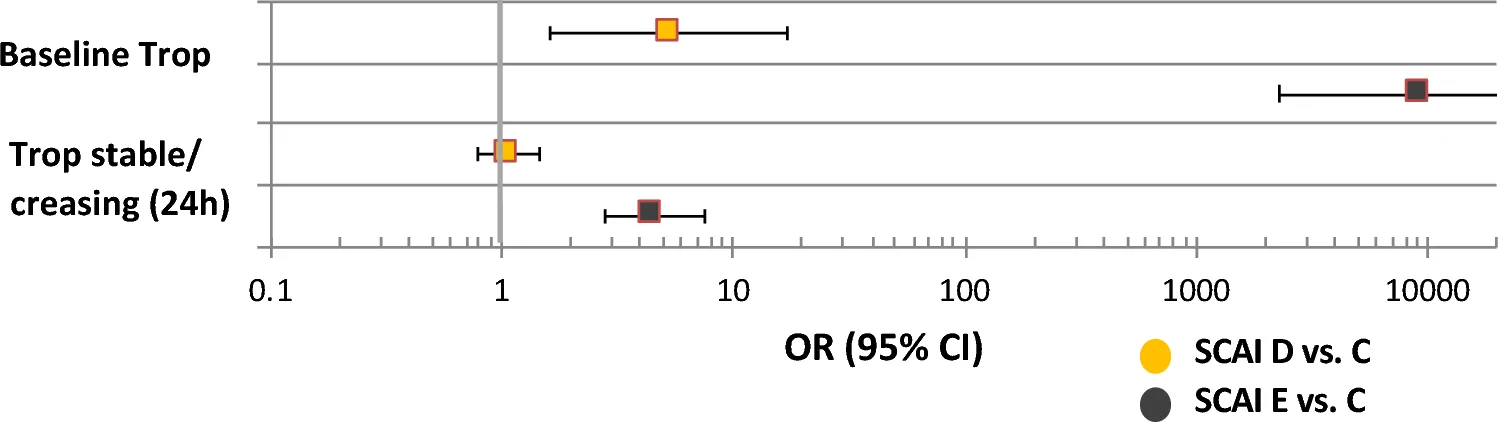
Troponin at baseline (per 250 ng/l increase) and increasing Troponin during the first 24 h of treatment associate with SCAI stages. Logistic regression model with adjustment for age, sex, baseline lactate, baseline pH, prior cardiac arrest incl. duration of CPR, acute on chronic vs. de-novo heart failure, mechanical ventilation and baseline creatinine. Trop: Troponin T measured by Elecsys® Troponin T-high sensitive assay (Roche). Increase/decrease of Troponin is defined as ≥ 20% change from baseline during the first 24 h. SCAI: cardiogenic shock classification by the Society for Cardiovascular Angiography and Interventions, ranging from A (at risk) to E (in extremis). OR (95% CI): Odds Ratio (95% Confidence Interval)

Troponin at baseline (shown per 250 ng/l increase) was associated with a higher likelihoods of presentation with SCAI stage D versus C (Odds Ratio [OR] 5.268, 95% confidence interval [CI]: 1.637, 16.953, *p* = 0.005) and E versus C (OR 9.313, 95% CI: 2.290, 37.878, *p* < 0.001) (Fig. 1). On the other hand, patients with SCAI stage E at baseline exhibited an increased probability of stable or increasing Troponin during the first 24 h vs. stage C (OR 4.503, 95% CI: 2.814, 7.579, *p* < 0.001).

### Troponin and mortality

**Fig. 2:**
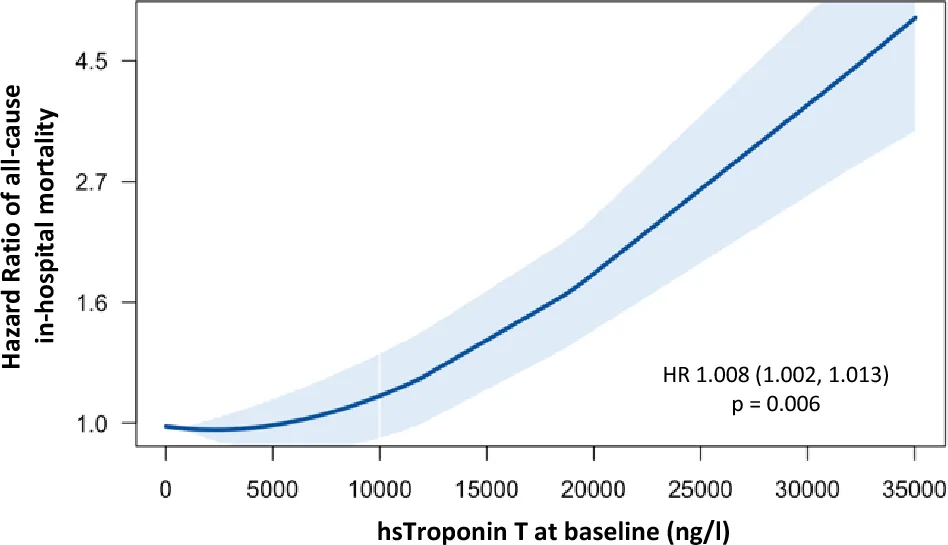
Troponin at baseline is a predictor of all-cause in-hospital mortality. Cox regression model with adjustment for age, sex, baseline lactate, baseline pH, prior cardiac arrest incl. duration of CPR, acute on chronic vs. de-novo heart failure, mechanical ventilation and baseline creatinine. hsTroponin T: Troponin T measured by Elecsys® Troponin T-high sensitive assay (Roche). HR: Hazard Ratio (95% Confidence Interval) (displayed per 250 ng/l Troponin increase)

**Fig. 3:**
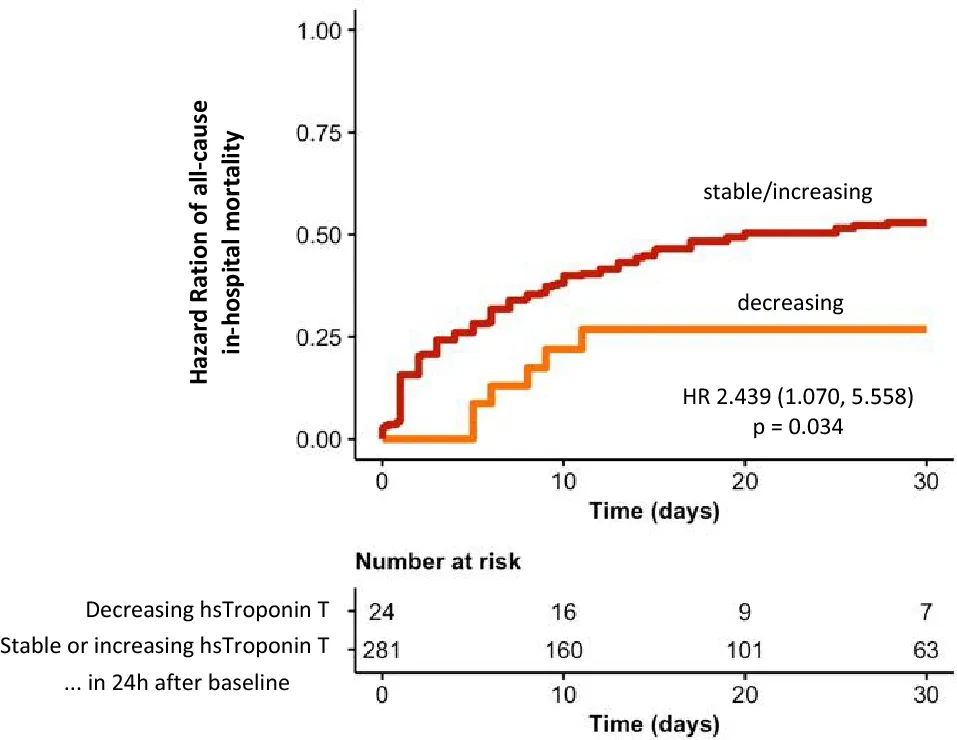
Troponin T kinetics within the first 24 h of treatment predict all-cause in-hospital mortality. Cox regression model with adjustment for age, sex, baseline lactate, baseline pH, prior cardiac arrest incl. duration of CPR, acute on chronic vs. de-novo heart failure, mechanical ventilation and baseline creatinine. hsTroponin T: Troponin T measured by Elecsys® Troponin T-high sensitive assay (Roche). Increase/decrease of Troponin is defined as ≥ 20% change from baseline during the first 24 h. HR: Hazard Ratio (95% Confidence Interval)

During hospitalisation, 228 patients (47.8%) died from any cause within 30 days. Troponin at baseline was significantly associated with in-hospital mortality after adjustment for confounders – age, sex, lactate, pH, prior cardiac arrest, acute-on-chronic vs. de-novo heart failure, LVEF, and creatinine – with a Hazard Ratio [HR] of 1.008 (95% CI: 1.002, 1.013, *p* = 0.001), narrowly missing significance after additional adjustment for centre in a sensitivity analysis (HR 1.005, 95% CI: 0.999–1.011; *p* = 0.088); all per 250 ng/l increase. No ceiling of this association could be detected. Results of the main analysis are shown as a spline chart in Fig. 2. Troponin T kinetics during the first 24 h after baseline proved to be a potent predictor of in-hospital mortality: Patients with stable or increasing Troponin exhibited a more than doubled risk of death compared to those with decreasing Troponin levels (HR 2.439, 95% CI: 1.070, 5.558, *p* = 0.034). Results are displayed as a Kaplan–Meier curve in Fig. 3.

## Discussion

This study assessed Troponin’s predictive capability for in-hospital mortality in a retrospective multinational cohort of consecutive patients exhibiting cardiogenic shock without acute myocardial infarction. Troponin at baseline was identified as a potent predictor of mortality. In addition, increasing Troponin kinetics within the first 24 h was significantly associated with higher mortality. In addition, higher Troponin at baseline correlated with the increased probability of a patient presenting with higher SCAI stages; stage E, vice versa, was associated with a greater likelihood of increasing Troponin kinetics.

### Troponin: a predictor established in myocardial infarction patients

Troponin is an integral element of cardiomyocytes, expressed almost exclusively in cardiac tissue, and increased levels indicate myocardial injury [14, 15]. In patients with AMI, Troponin levels correlate with the extent of myocardial necrosis [16, 17]. Thus, Troponin’s usability as a marker of risk stratification has been investigated previously in patients with AMI: In patients without ST-elevation, the higher the Troponin levels are, the greater is the risk of death [18, 19]. Routine Troponin measurement for prognostic reasons is not recommended, though, as it was shown not to improve patient management on top of, e.g., the GRACE score [20, 21]. The latter one is also recommended for prognostic evaluation of those patients presenting with ST-elevation [22]. Nevertheless, higher Troponin levels in patients with an AMI are associated with a higher probability of evident CS [23]. Other established biomarkers of use for prognostic stratification are, e.g., transaminase and creatinine levels [24, 25].

### Troponin’s predictive value in heart failure-related cardiogenic shock patients

Troponin levels above the 99th percentile, but without other criteria for acute myocardial infarction, are defined as myocardial injury [14]. Such Troponin elevations are frequent in the setting of heart failure [26–28]. They are likely caused by factors such as, e.g., wall stretch, inflammation, circulating neurohormones and exocytosis of cytosolic Troponin due to cardiomyocyte stress [14, 26, 29]. These factors also contribute to elevated Troponin levels in AMI-CS, even though the acute myocardial infarction remains the main determinant. In some patients, however, it remains challenging to differentiate isolated HF-CS from the extent of concomitant ischaemia. While ischaemia is clearly the underlying cause in patients with STEMI, its overlap with HF may be more difficult to assess in others. Essential clinical evaluations such as echocardiography and, when appropriate, coronary angiography can assist in this distinction. In the present study, experienced investigators at tertiary care centres excluded acute myocardial infarction as the cause of cardiogenic shock. However, this approach did not involve central adjudication and did not entirely rule out coexisting ischaemia – a limitation that should be considered when interpreting the findings.

Unlike in the setting of acute myocardial infarction and consequent cardiogenic shock, Troponin’s relevance in HF-CS patients regarding clinical outcomes remains rather unclear, as scientific evidence is scarce. Furthermore, most risk prediction tools were designed and evaluated in AMI-CS patients only; the CardShock score, as well as the CSS are exceptions [12, 13]. The present study intended to contribute to filling this gap in evidence.

### What’s new in this study?

There is an unmet need for improved mortality prediction in HF-CS patients. In accordance with the findings of the present study, Troponin could be used as a marker of myocardial injury: its baseline level and its kinetics are related to in-hospital mortality. It also correlates with the SCAI classification—emphasising that higher Troponin levels are associated with the severity of cardiogenic shock. While this study assessed high-sensitive Troponin T measured by Elecsys® Troponin T-high sensitive assay (Roche, Basel/Switzerland), the findings are likely to apply also to other Troponin subunits and assays as the underlying pathophysiological mechanism are the same.

Improved risk stratification is supposed to enhance patient management and, consequently, clinical outcomes. The same is true for (surrogate) parameters reflecting myocardial stress. This makes respective scores or, even simpler, circulating biomarkers measured in routine clinical care of high interest for clinicians. As a marker of myocardial injury, Troponin is an obvious candidate in this regard. Studies showed, though, that continuous Troponin did not add further value to patient management in patients with acute myocardial infarction beyond the well-established GRACE score with dichotomised Troponin (elevated vs not elevated) [20]. Nevertheless, Troponin could prove more valuable in the HF-CS setting: First, there is no such score that is both widespread and recognised for risk prediction. Second, HF-CS is a more heterogeneous condition than AMI-CS—especially with regards to cause and treatment. And, third, while Troponin is an excellent marker for myocardial infarction in AMI-CS patients, it is likely to reflect cardiogenic shock as a condition of its own better in HF-CS ones. Other biomarkers like lactate, also a surrogate parameter of cardiogenic shock severity, will remain of major importance, however. Integration of Troponin in established risk prediction scores should be assessed. Nonetheless, translating risk stratification into clinical decision-making and improved outcomes remains challenging, particularly given the limited high-quality evidence available for the treatment of HF-CS.

### Limitations

The following limitations should be considered when interpreting the results of this study:

First and foremost, by design, this study was not able to prove causal relationships but only show associations. However, this allows for hypothesis generation which may be useful for future randomised controlled trials.

Second, as variables for regression analyses were chosen in retrospect, the study is prone to confounding. There could be further relevant variables that were not considered.

Third, the definition of cardiogenic shock was left to the local investigators’ discretion. This may have resulted in different patient selection processes at different sites.

Similarly, the approach to exclude acute myocardial infarction and especially NSTEMI’s of the type 2 (AMI-CS vs. to HF-CS) may have been different between the sites, and retrospective validation is not feasible due to anonymisation of the data. However, all sites are experienced with treating cardiogenic shock and acute myocardial infarction. Thus, it can be assumed that diagnoses were compliant with the respective guidelines. Nonetheless, even in the absence of acute myocardial infarction, coexisting ischaemia may be present as well in patients with HF-CS. The study was not sufficiently powered to assess only the subgroup of patients without ischaemic cardiomyopathy, in whom NSTEMI type 2 would, however, still remain a possibility.

In addition, the timing of cardiogenic shock onset is likely to significantly influence both absolute troponin levels and their temporal dynamics. However, this information was not available in the database. A more detailed analysis of troponin kinetics in relation to the timing of shock onset, initiation of medical therapy, and the study-defined baseline would be of considerable interest but was not feasible due to the lack of corresponding data.

Finally, the additional prognostic value of Troponin to scores like, e.g., the CSS was not investigated as this study aimed rather at pathophysiological insights. Moreover, given the limited high-quality evidence for the treatment of HF-CS, the clinical relevance of improved risk stratification remains to be determined.

## Conclusions

Mortality in patients with cardiogenic shock not caused by acute myocardial infarction remains high and better risk stratification is urgently needed to improve clinical outcomes. Most established scores have been evaluated in patients with cardiogenic shock due to acute myocardial infarction. In this multinational study, higher baseline Troponin as well as increasing Troponin levels were associated with greater all-cause in-hospital mortality. In addition, Troponin associated with cardiogenic shock severity according to the SCAI classification. These data highlight Troponin’s potential to serve as a risk discriminator in patients with heart failure-related cardiogenic shock both for routine clinical care and for future clinical trials.

## Clinical Perspectives

### Clinical competencies

- Troponin is an established marker of acute myocardial infarction and, thus, in cardiogenic shock complicating acute myocardial infarction.
- Heart failure-related cardiogenic shock accounts for almost 50% of all cardiogenic shock cases, but is less well investigated.
- This paper adds evidence that Troponin also associates with shock severity and predicts mortality in patients with heart failure-related cardiogenic shock. This association remains significant even after adjustment for other clinically relevant parameters, including lactate.

### Translational outlook

- Assessing Troponin’s additional value when added to scores like the CSS may be evaluated to optimise risk prediction in patients with heart failure-related cardiogenic shock.
- In addition to optimal risk prediction, defining treatment allocation depending on the predicted risk at baseline is needed to actually improve outcomes.

## Supplementary Information

Below is the link to the electronic supplementary material.

Supplementary file1 (DOCX 17 kb)

## Abbreviations

AMI: Acute myocardial infarction
AMI-CS: Cardiogenic shock complicating acute myocardial infarction
CI: Confidence interval
CS: Cardiogenic shock
CSS: Cardiogenic Shock Score
ECG: Electrocardiogram
eCPR: Extracorporeal cardiopulmonary resuscitation
GRACE score: The Global Registry of Acute Coronary Events score
HF-CS: Heart failure-related cardiogenic shock
HR: Hazard Ratio
LVEF: Left ventricular ejection fraction
NSTEMI: Non-ST-elevation myocardial infarction
OR: Odds Ratio
SCAI: Cardiogenic shock classification by the Society for Cardiovascular Angiography and Interventions

## Declarations

### Data Availability

The datasets used and/or analysed during the current study are available from the corresponding author on reasonable request.
