# Supplementary material for: Troponin predicts mortality in heart failure-related cardiogenic shock
Source: Intern Emerg Med. 2025 Nov 18;21(8):3015–23. doi: 10.1007/s11739-025-04197-7 (PMC13751742; doi:10.1007/s11739-025-04197-7)
Supplement: Supplementary file 1 [file 11739_2025_4197_MOESM1_ESM.docx]

# Supplement

|  | **< median**  **N=236** | **≥ median**  **N=241** | **p-value** | **Missing**  **N (%)** |
| --- | --- | --- | --- | --- |
| **Demographical data** | | | | |
| **Age (years, mean (SD))** | 62.87 (15.03) | 62.03 (16.62) | 0.563 | 0 (0.0) |
| **Sex (female) (%)** | 63 (26.7) | 81 (33.6) | 0.122 | 0 (0.0) |
| **Heart failure characteristics** | | | | |
| **History of heart failure (%)** | 146 (62.1) | 109 (45.2) | < 0.001 | 1 (0.2) |
| **Ischaemic cardiomyopathy (%)** | 71 (62.8) | 64 (57.7) | 0.513 | 253 (53.0) |
| **LVEF (%, median (IQR))** | 20 (15, 30) | 20 (15, 30) | 0.083 | 51 (10.7) |
| **Resuscitation** | | | | |
| **CPR (%)** | 81 (34.3) | 115 (47.7) | 0.004 | 0 (0.0) |
| **Shockable rhythm (%)** | 46 (56.1) | 66 (59.5) | 0.749 | 284 (59.5) |
| **ROSC (min, median (IQR))** | 0.00 (0.00, 15.00) | 10.00 (0.00, 27.75) | < 0.001 | 160 (33.5) |
| **Haemodynamics** | | | | |
| **Systolic blood pressure (mmHg, mean (SD))** | 83.28 (17.23) | 80.83 (16.58) | 0.118 | 12 (2.5) |
| **Mean arterial pressure (mmHg, mean (SD))** | 59.67 (13.57) | 58.38 (12.13) | 0.309 | 60 (12.6) |
| **Heart rate (bpm, median (IQR))** | 114.00 (90.00, 134.50) | 100.00 (80.00, 125.00) | 0.001 | 3 (0.6) |
| **Vasopressors (%)** | 218 (92.4) | 228 (94.6) | 0.422 | 0 (0.0) |
| **Use of any MCS (%)** | 124 (52.5) | 114 (47.3) | 0.292 | 0 (0.0) |
| **Laboratory** | | | | |
| **pH (median (IQR))** | 7.30 (7.20, 7.38) | 7.27 (7.13, 7.35) | 0.004 | 8 (1.7) |
| **Lactate (mmol/l, median (IQR))** | 7.55 (4.47, 10.00) | 6.90 (3.95, 11.38) | 0.911 | 23 (4.8) |
| **Creatinine (mg/dl, median (IQR))** | 1.73 (1.32, 2.46) | 1.80 (1.33, 2.70) | 0.240 | 2 (0.4) |
| **hsTroponin T (ng/l, median (IQR))** | 75.50 (41.75, 104.25) | 677.00 (325.00, 1560.00) | < 0.001 | 0 (0.0) |
| **SCAI at enrolment** | | | | |
| **SCAI A (%)** | 0 (0) | 0 (0) | < 0.001 | 0 (0.0) |
| **SCAI B (%)** | 0 (0) | 0 (0) |  |  |
| **SCAI C (%)** | 132 (55.9) | 106 (44.0) |  |  |
| **SCAI D (%)** | 77 (32.6) | 70 (29.0) |  |  |
| **SCAI E (%)** | 27 (11.4) | 65 (27.0) |  |  |
| **Triggers of shock** | | | | |
| **Tachyarrhythmia (%)** | 91 (38.7) | 46 (22.2) | < 0.001 | 35 (7.3) |
| **No trigger identified (%)** | 57 (24.3) | 73 (35.3) |  |  |
| **Infection (%)** | 48 (20.4) | 42 (20.3) |  |  |
| **Stress (%)** | 8 (3.4) | 24 (11.6) |  |  |
| **Non-adherence (%)** | 9 (3.8) | 7 (3.4) |  |  |

Supplemental Table 1: Patient characteristics stratified by Troponin level at baseline. < median: patient with baseline Troponin below the median. ≥ median: patients with baseline Troponin equal to or higher than the median. LVEF: left ventricular ejection fraction. CPR: cardiopulmonary resuscitation. ROSC: return of spontaneous circulation. MCS: mechanical circulatory support devices. hsTroponin T: Troponin T measured by Elecsys® Troponin T-high sensitive assay (Roche). SCAI: cardiogenic shock classification by the Society for Cardiovascular Angiography and Interventions, ranging from A (at risk) to E (in extremis). SD: standard deviation. IQR: interquartile range. Relative frequencies calculated excluding missing values.

| Time of measurement | Troponin, median (IQR) | Missing, N (%) |
| --- | --- | --- |
| Day 0 | 164.00 (77.00, 678.00) | 0 (0.0) |
| Day 1 | 399.50 (108.75, 1277.50) | 195 (40.9) |
| Day 3 | 231.00 (73.00, 784.00) | 368 (77.1) |
| Day 5 | 164.00 (56.00, 525.00) | 392 (82.2) |
| Day 7 | 168.00 (59.00, 470.00) | 410 (86.0) |

Supplemental Table 2: Serial Troponin measurements. hsTroponin T: Troponin T measured by Elecsys® Troponin T-high sensitive assay (Roche). IQR: interquartile range.
